# Quantitative Estimation of Cell-Associated Silver Nanoparticles using the Normalized Side Scattering Intensities of Flow Cytometry

**DOI:** 10.3390/nano11113079

**Published:** 2021-11-15

**Authors:** Yoo Jin Shim, My Kieu Ha, Tae Hyun Yoon

**Affiliations:** 1 Department of Chemistry, College of Natural Sciences, Hanyang University, Seoul 04763, Korea; youckdduf@gmail.com (Y.J.S.); hakieumy12@gmail.com (M.K.H.); 2 Institute of Next Generation Material Design, Hanyang University, Seoul 04763, Korea; 3 Research Institute for Convergence of Basic Science, Hanyang University, Seoul 04763, Korea

**Keywords:** cellular association of nanoparticles, flow cytometry, silver nanoparticles

## Abstract

Quantification of cellular nanoparticles (NPs) is one of the most important steps in studying NP–cell interactions. Here, a simple method for the estimation of cell-associated silver (Ag) NPs in lung cancer cells (A549) is proposed based on their side scattering (SSC) intensities measured by flow cytometry (FCM). To estimate cellular Ag NPs associated with A549 cells over a broad range of experimental conditions, we measured the normalized SSC intensities (nSSC) of A549 cells treated with Ag NPs with five different core sizes (i.e., 40–200 nm, positively charged) under various exposure conditions that reflect different situations of agglomeration, diffusion, and sedimentation in cell culture media, such as upright and inverted configurations with different media heights. Then, we correlated these nSSC values with the numbers of cellular Ag NPs determined by inductively coupled plasma mass spectrometry (ICPMS) as a well-established cross-validation method. The different core sizes of Ag NPs and the various exposure conditions tested in this study confirmed that the FCM-SSC intensities are highly correlated with their core sizes as well as the amount of cellular Ag NPs over a linear range up to ~80,000 Ag NPs/cell and ~23 nSSC, which is significantly broader than those of previous studies.

## 1. Introduction

Nanoparticles (NPs) have been widely used in various consumer products, such as cosmetics, medicine, textiles, and sporting equipment [1,2,3]. To achieve a systematic understanding of the interactions of NPs with biological systems, it is necessary to develop simple, easy, and quantitative measurement methods for cellular NPs. Currently, the number of cellular NPs can be measured by either direct (e.g., fluorescence or electron microscopy) [4] or indirect (e.g., inductively coupled plasma mass spectroscopy (ICPMS)) analytical techniques [5,6]. However, quantification using these approaches is limited by difficulties in fluorescence signal calibrations (e.g., fluorescence microscopy) and collecting a sufficient number of representative images (e.g., transmission electron microscopy) [7]. ICPMS measurements can also be performed in parallel with other biological assays to measure the number of cellular NPs, but this method requires additional sample preparation procedures with labor-intensive and time-consuming steps.

The side scattering (SSC) signal of flow cytometry (FCM) has been applied to estimate cellular NPs, since it is known to reflect the inner complexity or granularity of cells [8,9,10,11,12]. Therefore, these light-scattering signals, particularly SSC, from FCM can offer valuable insight into the interactions between biological cells and NPs. For instance, it was previously reported that SSC intensity is closely related to the number of cellular NPs [9,10,11,12]. However, the cellular behavior of nanoparticles is known to be complex and heterogeneous, partially due to the diffusion, sedimentation, and agglomeration of NPs in cell culture media. This can be affected by many biological and physicochemical factors, such as the type of cell line [13] and NPs [14,15,16,17,18]. For instance, the type, size, and shape of NPs may influence the transport processes of NPs in culture media, typically diffusion and sedimentation, and may result in differences in their cellular associations. Previously, the importance of sedimentation and diffusion of NPs was recognized, and particokinetic models were proposed by Teeguarden and Hinderliter [14,15] to calculate the effective dose of NPs for in vitro systems. Moreover, DeLoid et al. [16,17,18] reported a particokinetic model for the agglomeration of NPs, which seems to have an important role in determining the fate and transport of NPs. Additionally, the variations in the type, size, and shape of cells and their subcellular organelles caused by differences in their growth/exposure conditions may also result in differences in their SSC intensity and cause errors in estimating cellular NPs. Considering these numerous factors affecting the cell–NP interaction, it is important to perform studies under various experimental conditions. In this study, to estimate cellular silver NPs associated with A549 cells, we measured the normalized SSC intensities (nSSC) of A549 cells exposed to Ag NPs with five different core sizes and a positively charged surface. Then, nSSC values were correlated with the concentrations of cellular silver NPs confirmed by inductively coupled plasma mass spectrometry (ICPMS). Additionally, we also tested various exposure conditions, such as upright and inverted configurations with media heights of 3, 6, and 9 mm. Ag NPs were chosen in this study, since they are among the most widely used NPs due to their strong antibacterial and antifungal abilities [19,20,21]. Upright and inverted exposure configurations with different media heights of 3, 6, and 9 mm were used to test biological and physicochemical conditions facilitating different transport processes (sedimentation and diffusion) of NPs in the cell culture medium.

## 2. Materials and Methods

### 2.1. Silver Nanoparticles

In this study, we used Ag NPs (BioPure Silver Nanoparticles, Nanocomposix, San Diego, CA, USA) with nominal diameters of 40, 60, 80, 100, and 200 nm capped with positively charged branched polyethlyeneimine (bPEI). The 5 types of Ag NPs are abbreviated Ag^40^, Ag^60^, Ag^80^, Ag^100^, and Ag^200^.

### 2.2. Physicochemical Characterization of Ag NPs

Ag NP dispersions were prepared in deionized (DI) water and RPMI-1640 (Roswell Park Memorial Institute) media supplemented with 10% FBS (fetal bovine serum) and 1% penicillin–streptomycin. A particle size analyzer (Zetasizer Nano-ZS, Malvern Instruments Ltd., Malvern, UK) was used to measure the hydrodynamic sizes and surface charges of Ag NPs. UV–vis absorbance, measured with a spectrophotometer (Mecasys Optizen– 2120UV, Daejeon, Korea), at 24 h was divided by absorbance at 0 h to determine the dispersion stability.

### 2.3. Cell Culture and Ag NP Exposure

**Figure 1:**
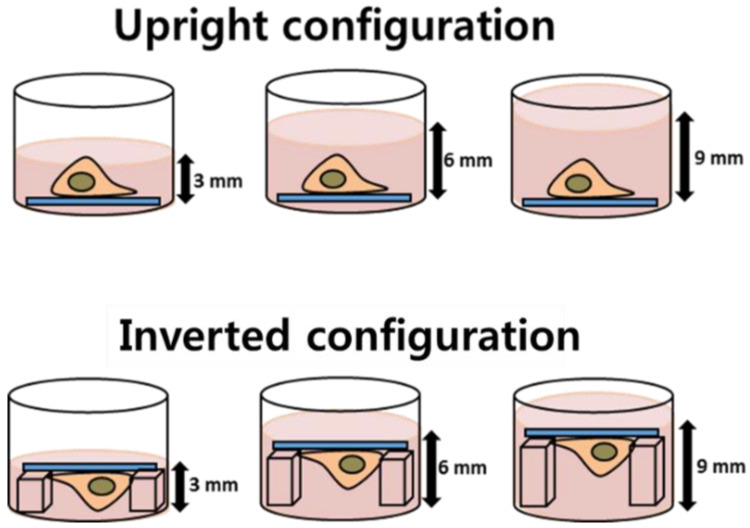
Illustration of cell configurations (upright and inverted) with different media heights: 3, 6, and 9 mm.

The A549 cell line (CCL-185, ATCC, Manassas, VA, USA) was obtained from the KCLB (Korea Cell Line Bank, Seoul, Korea) and cultured in fresh RPMI-1640 media. Cells were seeded on coverslips treated for tissue culture (25 mm in diameter, Nunc^TM^ Thermanox^TM^, Thermo Fisher Scientific, Waltham, MA, USA), which were placed in each well of 6-well tissue culture plates (SPL Life Sciences, Gyeonggi-do, Korea). After seeding, cells were allowed to adhere overnight in an incubator (Forma Scientific, Marietta, OH, USA) at 37 °C and 5% CO_2_. In the upright configuration, the coverslips were placed at the bottom of a well with adherent cells facing up, while the coverslips in the inverted configuration were placed on top of two polydimethylsiloxane (PDMS) blocks of different heights: 3, 6, and 9 mm, with adherent cells facing down, as illustrated in Figure 1. Next, 10 µg/mL dispersions of Ag NPs were filled to heights of 3, 6, or 9 mm and treated for 24 h before measurements.

### 2.4. Cell Culture and Ag NP Exposure 

For flow cytometry measurements, after exposure to Ag NPs, the cells were washed three times with Dulbecco’s phosphate-buffered saline (DPBS, Welgene, Geyongsangbuk-do, Korea), trypsinized, centrifuged at 3000 rpm (201 g) for 1 min, and resuspended in DPBS. These cells were analyzed using a flow cytometer (Accuri C6, BD Biosciences, Franklin Lakes, NJ, USA) equipped with a 488 nm argon laser. FCS Express 6 Plus (De Novo Software, Los Angeles, CA, USA) was used to analyze the FCM data.

### 2.5. ICPMS Measurement

To confirm the numbers of cellular Ag NPs, ICPMS (NexION 300D, PerkinElmer, Inc., Waltham, MA, USA) measurements were conducted and compared with the results of flow cytometry. Resuspended cells were counted with a hemocytometer and digested with conc. nitric acid (65%). Samples were then diluted in 3% nitric acid before ICPMS measurements. Standard solution of Ag^+^ ions (NexION, PerkinElmer, Inc., Waltham, MA, USA) was used to prepare a calibration curve for quantification.

### 2.6. Data Analysis

FCM and ICPMS results were compared to develop equations for the estimation of cellular Ag NPs. By dividing the SSC intensity of each sample with the mean SSC intensity of the control sample, SSC intensities of each sample were normalized (nSSC = (SSC)_i_⁄(SSC)_o_, nSSC = normalized SSC intensity, (SSC)_i_ = mean SSC intensity of cells exposed to Ag NPs, (SSC)_o_ = mean SSC intensity of control cells). A training dataset including nSSC values, core diameters of Ag NPs, and cell-associated Ag NPs in mass-based and number-based units was used to develop the model equations. A separate dataset was used to validate the model. The cellular Ag NP values (c-Ag NPs) determined by ICPMS were compared with the c-Ag NP values estimated from the model equations and the nSSC values measured by FCM.

## 3. Results

### 3.1. Physicochemical Characteristics of Ag NPs

The physicochemical properties of Ag NPs used in this study were thoroughly characterized and are listed in Table 1. The hydrodynamic sizes of Ag NPs in serum-containing RPMI-1640 media were larger than those in deionized (DI) water, which was likely because of the adsorption of serum proteins onto the surfaces of Ag NPs [22,23] and agglomeration caused by interaction between surrounding proteins. In RPMI-1640, hydrodynamic sizes at 24 h were typically larger than those at 0 h. This might be caused by an increase in particle agglomeration over time. Ag^40^ NPs showed the most dramatic increase in hydrodynamic size, while the sizes of other NPs did not change significantly. These observations indicate that small NPs agglomerated more than large NPs, which is reasonable, as there are more small NPs per unit volume than large NPs in a dispersion, given the same mass concentration [24]. All highly positive zeta potentials of Ag NPs in DI water changed to slightly negative values of approximately –10 mV in RPMI-1640 media. This phenomenon was also likely caused by the adsorption of serum proteins. Proteins are known to have a slight negative charge [24], so they may have formed protein coronas and changed the surface charge of Ag NPs.

In DI water, most dispersion stability values were close to 1, indicating that these dispersions were quite stable with little sedimentation even after 24 h, while in RPMI-1640 media, the dispersions became less stable, and their dispersion stability values decreased as nominal size increased, except for Ag^40^ NPs. This unexpected instability of the Ag^40^ NP dispersion may be related to the unusual agglomeration of Ag^40^ NPs. As can be seen in Table 1, Ag^40^ NPs experienced the greatest size increase in culture media (3.37-fold increase between the hydrodynamic size and nominal size), whereas other Ag NP sizes in this study only had around a 2-fold increase. This result also indicates that unusually high agglomeration occurred for Ag^40^ NPs.

When exposed to A549 cells in RPMI-1640 media, the fates of Ag NPs are directed by sedimentation and diffusion processes, which are known to be strongly influenced by the physicochemical properties of Ag NPs, such as hydrodynamic size, surface charge, and dispersion stability [25,26]. These sedimentation and diffusion processes also determine the effective dose of NPs and their cellular associations. Therefore, we conducted experiments using upright and inverted configurations with different media heights to test the effects of the various physicochemical properties described in this section, as well as sedimentation and diffusion processes.

### 3.2. Cellular Ag NPs Measured by FCM and ICPMS

In flow cytometry, SSC intensity is related to the internal complexities of cells and, therefore, can be used to estimate the cellular association of NPs. As described previously by Suzuki et al. [9], the FCM-SSC method has been applied in several studies to qualitatively estimate the cellular association of NPs in mammalian cells. However, to quantitatively measure the number of cellular NPs, it is necessary to conduct parallel experiments (e.g., ICPMS) along with FCM measurements for biological assays. Following our recent studies on the estimation of cellular SiO_2_ and Au NPs by FCM-SSC [8,27], we expanded the application of this simple method to estimate the cellular uptake of Ag NPs in the A549 cell line by employing the SSC intensity of flow cytometry and providing equations for their relationships with the number of cellular NPs.

In the proposed method, in addition to FCM, we used ICPMS to measure the number of cellular Ag NPs. The SSC intensity from FCM was compared with the number of cellular Ag NPs measured by ICPMS. The relationship shown by these results is represented in equations that can be used to estimate cellular Ag NPs by placing the SSC intensity of cells into the equation; therefore, after the equation is established, there is no need to perform additional ICPMS measurements. It is important to note that the ICPMS measurement in this study is a calibration procedure, which provides input quantities, thus allowing quantification.

**Figure 2:**
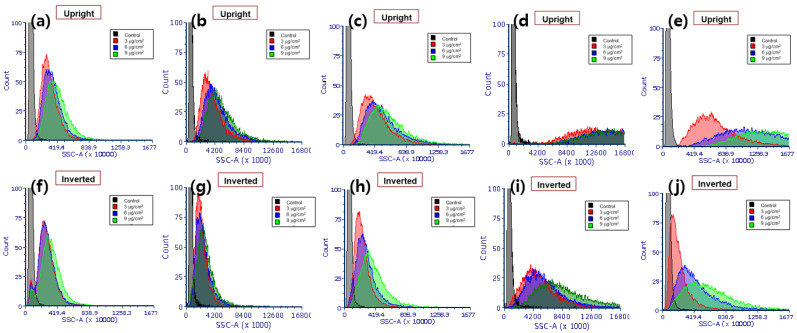
FCM-SSC intensity histograms of A549 cells exposed to (**a**,**f**) Ag^40^, (**b**,**g**) Ag^60^, (**c**,**h**) Ag^80^, (**d**,**i**) Ag^100^, and (**e**,**j**) Ag^200^ NPs under upright (**a**–**e**) and inverted (**f**–**j**) configurations.

**Figure 3:**
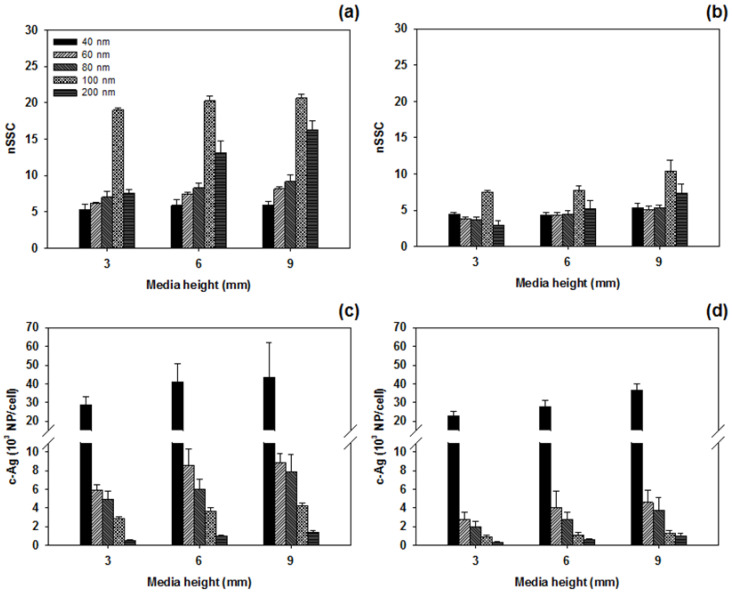
(**a**,**b**) Normalized side scattering intensity and (**c**,**d**) cellular association of Ag NPs against media height in (**a**,**c**) upright and (**b**,**d**) inverted configurations. Error bars indicate standard deviation of 3 replicate measurements.

Figure 2 shows the SSC intensities of A549 cells in upright and inverted configurations exposed to 10 μg/mL Ag NPs with different core sizes (40, 60, 80, 100, and 200 nm) in media with three different heights (3, 6, and 9 mm). To compensate for signal fluctuations in different sample batches, the SSC intensities of cells exposed to Ag NPs were normalized to those of control cells, as shown in Figure 3a,b. The number of cell-associated Ag NPs per cell measured by ICPMS is presented in Figure 3c,d. The SSC intensities showed significant changes with respect to the core sizes, media heights of Ag NPs, and cell configurations. Except for the abnormal decrease observed for NPs with the largest diameter (i.e., Ag^200^), the SSC intensity generally increased when cells were exposed to larger Ag NPs in the upright configuration. However, the SSC intensity of cells in the inverted configuration showed no clear patterns with respect to particle size. In contrast, the ICPMS results showed that the number of cell-associated Ag NPs in both configurations decreased as particle size increased. The different tendencies in the FCM and ICPMS results may be related to the particle size dependence of the scattered light. The intensity of scattered light is known to be proportional to the diameter of the particle [6,28]. Thus, cells exposed to larger Ag NPs had higher SSC intensities than those exposed to smaller Ag NPs, although small Ag NPs showed more associations with cells than larger Ag NPs. In addition to the size dependence, there were also signs of media height dependence. At the same administered dose of 10 μg/mL, different media heights may lead to differences in the total number of particles available to transport to cells. Thus, as media height increased, both SSC intensity and the cellular association of Ag NPs increased. Between the two configurations, SSC intensities and the number of cell-associated Ag NPs in the upright configuration were higher than those in the inverted configuration. This may be because in the upright case, particle transport occurred by both sedimentation and diffusion, while in the inverted case, transport occurred only by diffusion. Therefore, upright cells may associate with more Ag NPs than inverted cells. The ICPMS results revealed that the number of cell-associated Ag^40^ NPs was unexpectedly high. This is likely because of the hydrodynamic size of Ag^40^ NPs. As shown in Table 1, Ag^40^ NPs showed a larger increase in hydrodynamic size from DI water to RPMI-1640 media than other sizes. This may be accompanied by higher degrees of protein adsorption and receptor–ligand interactions, which enhance receptor-mediated endocytosis [29].

In culture medium, there is competition for cellular receptors between protein–NP complexes and free proteins.^17^ A larger amount of protein adsorbed onto the surfaces of Ag^40^ NPs, indicating that there is a small amount of free protein in the culture medium; therefore, the opportunities of protein–NP complexes to interact with cellular receptors are higher for Ag^40^ NPs than other particles. Additionally, a higher number of cell-associated Ag^40^ NPs might indicate a substantial agglomeration of NPs due to their larger surface areas [30].

### 3.3. nSSC-Based Estimation of Cell-Associated Ag NPs

**Figure 4:**
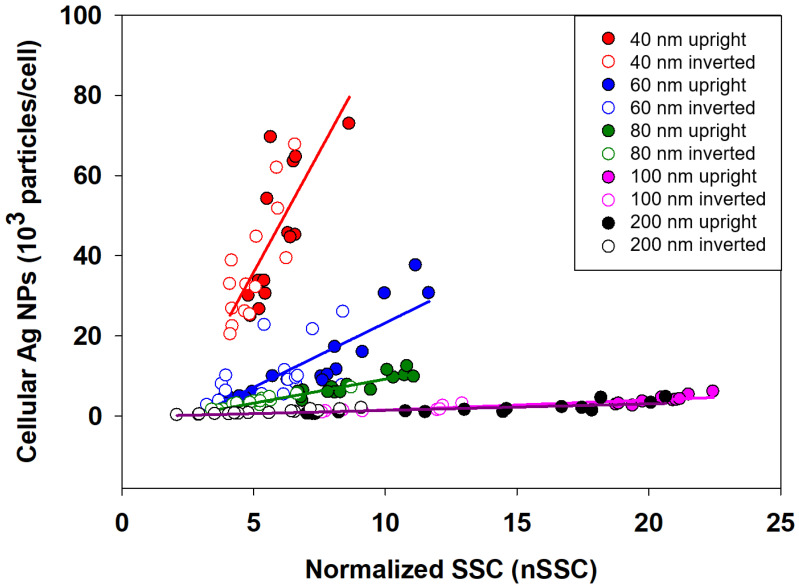
Linear regression between number of cellular Ag NPs and normalized SSC for 40, 60, 80, 100, and 200 nm particle sizes in upright and inverted configurations.

The normalized SSC (nSSC) intensities were linearly fitted with the number of cellular Ag NPs based on the presumption that nSSC has a unity value for the control sample (i.e., nSSC = 1 when c-Ag = 0). The regression result is plotted in Figure 4. Similar to previous results for Au NPs in HeLa cells [8], linear equations with distinct slopes were found for each particle size. The slopes of the equations decrease as the core diameters of Ag NPs increase (Equations (1)–(5) for Ag^40^, Ag^60^, Ag^80^, Ag^100^, and Ag^200^ NPs, respectively), which agrees well with observations in previous studies [8,27]. For the smaller Ag NPs (i.e.., Ag^40^, Ag^60^, and Ag^80^), the slope is highly sensitive to the core size, while it becomes less sensitive to the core size of Ag NPs larger than 100 nm.

c-Ag (no./cell) = 9423 × (nSSC − 1), R^2^ = 0.9457 (1)

c-Ag (no./cell) = 2301 × (nSSC − 1), R^2^ = 0.8429 (2)

c-Ag (no./cell) = 967 × (nSSC − 1), R^2^ = 0.9683 (3)

c-Ag (no./cell) = 204 × (nSSC − 1), R^2^ = 0.9511 (4)

c-Ag (no./cell) = 159 × (nSSC − 1), R^2^ = 0.8571 (5)

The slopes in each equation were then fitted with the core diameters of Ag NPs (Equation (6)) and merged with the above equations to generate a combined equation (Equation (7)) for number-based cellular Ag NPs:

Slope = 199 + 161933 e^−0.072(Core diameter)^ (6)

c-Ag (no./cell) = (199 + 161933 e^−0.072(Core diameter)^) (nSSC − 1) (7)

These equations indicate that there is a strong correlation among SSC intensities, core diameter of Ag NPs, and cellular uptake. These empirical equations are in accordance with formal light-scattering theories (Rayleigh and Mie scattering theories) in the sense that light-scattering behavior can be affected by particle size. In Rayleigh and Mie scattering theories, the intensity of the scattered light is proportional to d^6^ (d—particle diameter) and 1/r^2^, respectively. However, in the empirical equation obtained in this study, the relationship between light scattering and particle size was not in accordance with either theory. This result is understandable, as a biological system is complicated; thus, at the current stage, it is difficult to explain the correlation with a specific light-scattering theory. Thus, in this study, the equations were merely based on the observation and statistical treatment of the obtained experimental data.

**Figure 5:**
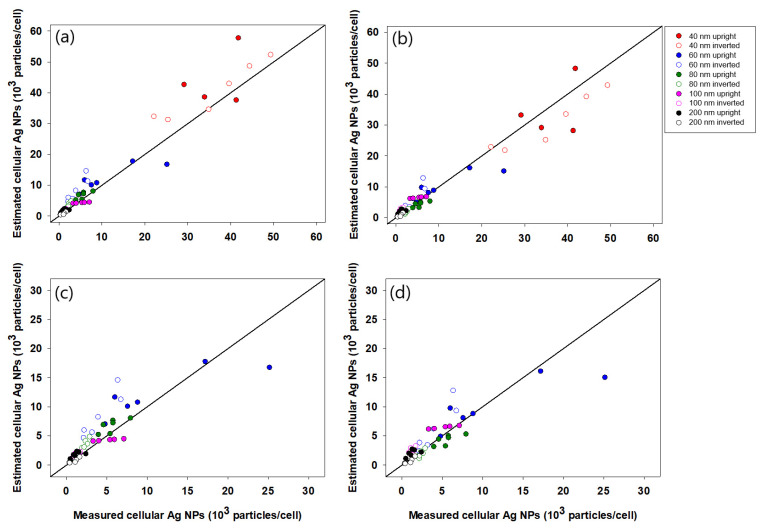
Validation of model equations. Measured number of c-Ag NPs was compared with estimated values using (**a**,**c**) separate equations for each particle size and (**b**,**d**) one equation incorporating particle size. Panels (**a**) and (**b**) show validation results for all 5 particle sizes, whereas panels (**c**) and (**d**) exclude Ag^40^ NPs.

To validate the linear regression equations, nSSC values from the validation dataset were used to estimate the number of cellular Ag NPs, and the estimation results were then compared with cellular Ag NPs measured by ICPMS (see Figure 5). The parameter root-mean-squared error (RMSE) was used to assess the extent to which the estimated cellular Ag NPs deviated from the values measured by ICPMS. Lower RMSE indicates less deviation and better agreement between estimated and measured values. As shown in Figure 5a, the number of cellular Ag NPs was estimated by individual equations for different particle sizes (i.e., Equations (1)–(5)), where the RMSE value was 387. However, as shown in Figure 5b, when the number of cellular Ag NPs was estimated using a single equation involving the core sizes of nanoparticles (i.e., Equation (7)), the value of RMSE increased to 3464, which is nearly 10-fold higher than that shown in Figure 5a. These results suggest that the estimation of number-based cellular Ag NPs based on individual equations for different particle sizes showed better performance than the estimation using a single equation involving the core size of NPs.

Although Figure 5a,b shows a relatively good correlation, data from Ag^40^ NPs demonstrated significant deviation from the diagonal line, which reflects complete agreement between the measured and estimated number of cell-associated Ag NPs. The deviation leads us to assume that data from Ag^40^ NPs are outliers, which should not be used for validating the performance of the linear regression equation. Additionally, the assumption that data for Ag^40^ NPs are outliers is also supported by the specific characteristics shown by Ag^40^ NPs, such as unusually larger hydrodynamic sizes in RPMI-1640 media and higher cellular association compared to other Ag NPs. Therefore, based on this assumption, we attempted additional validation with the exclusion of data for Ag^40^ NPs to determine whether this would improve the validation result. When data for Ag^40^ NPs were excluded, the estimated number of cellular Ag NPs showed better agreement with the quantity measured by ICPMS. When individual equations for different-sized NPs (i.e., Equations (1)–(5)) were used, the value of RMSE was significantly reduced from 387 to 32, whereas the value of RMSE decreased from 3464 to 2154 when the size-incorporated single equation (i.e., Equation (7)) was used. This finding indicates that the estimation model for number-based cellular Ag NPs performed better for Ag NPs with cores sizes larger than 40 nm, which was very similar to the results for Au NPs associated with HeLa cells in our previous study [8].

## 4. Discussion

As described above, the unique characteristics of side-scattered light signals from FCM measurements can provide a simple and valuable method to quantify the cellular association of NPs. Recently, we combined FCM-SSC intensity measurements with those of ICPMS and X-ray fluorescence to develop a method for assessing the number of cell-associated NPs (e.g., Au and SiO_2_ NPs) [8,27]. A similar approach was applied in this study for Ag NPs to develop a simple quantification method for the Ag NPs associated with A549 cells under various exposure conditions, i.e., cell culture configuration (upright and inverted) with different media heights, using the FCM and ICPMS techniques. Similar to the previous results for Au and SiO_2_ NPs, we found that the FCM-SSC intensities were closely related to the number of cellular Ag NPs and particle sizes, for which empirical equations reflecting the relationship among these three parameters are proposed, and the number of cellular Ag NPs can be estimated from the measurement of FCM-SSC intensities and the core size of Ag NPs provided by the manufacturer.

Although the equations proposed for Au, Ag, and SiO_2_ NPs were empirical (based on the statistical treatments of experimental observations), there are similarities between these empirically derived equations and fundamental scattering principles (e.g., Mie scattering theory), where the SSC intensities are dependent on the size, type, and number of NPs. On the other hand, there are also dissimilar features in the observed relationships (e.g., empirically determined coefficients of Equations (1)–(5) and (7)), which cannot be explained only by fundamental scattering principles. We suggest that these features are due to the biological (e.g., cell division and growth) complexities of the system. Thus, various exposure conditions, such as upright and inverted configurations with different media heights, were tested to confirm that the FCM-SSC intensities are not greatly influenced by these biological factors, such as variations in cellular size and shape and subcellular organelle size/shape, due to differences in growth/ exposure conditions.

Additionally, compared to previous studies, significant improvement in the linear range of cellular NP quantification was achieved in this study, nearly 20-fold greater than that in previous studies (see Table 2). For example, the linear range for the quantification of NPs was less than ~3500 Au NPs/cell and ~1.6 nSSC in a recent study by Park et al. [8]. In contrast, the linear range for the quantification of cellular NPs is up to ~80,000 Ag NPs/cell and ~23 nSSC, which can be mainly ascribed to the various experimental conditions adopted in this study, allowing us to collect wider ranges of data on cellular NPs. All of these observations suggest that the FCM-SSC-based method of cellular NP quantification can be more generally applied to various types of NPs, cell lines, and exposure conditions.

## 5. Conclusions

Here, based on the nSSC values measured by FCM, a simple method for the estimation of cell-associated silver (Ag) NPs in lung cancer cells (A549) is proposed. To estimate cellular Ag NPs associated with A549 cells, (1) the concentrations of cell-associated Ag NPs were determined by ICPMS, as a well-established cross-validation method, (2) SSC intensities were then measured for A549 cells exposed to various types of silver NPs and converted to normalized SSC (nSSC) values, and (3) the relationships between these measured values were found via statistical treatments of experimental observations. Through this procedure, we confirmed that the FCM-SSC intensities were closely related to the number of cellular Ag NPs and particle sizes, while they were not much affected by the other biological factors. Combined with our recent studies on Au and SiO_2_ NPs, it was also confirmed that this FCM-SSC-based method can be applied to various types of NPs and cell lines over a significantly wider linear range for Ag NPs.

However, we also recommend that more in-depth studies be performed for other NPs and cell lines under various exposure conditions, and careful analysis should be performed to determine whether the relationship between the nSSC and cellular NPs can be further generalized, rather than having empirical equations based on case-by-case observations. We also would like to emphasize that the proposed approach is in the early stage of analytical method development, rather than a commercialized protocol in practice at biological laboratories. Although we demonstrated a few important correlations among variables and provided insights into the cause-and-effect relationship based on fundamental scattering theory, further engineering studies are also needed to make this analytical method practically applicable in common biological laboratories. Further research directions can include the development of standard/reference beads containing known quantities of NPs, validation of empirical equations for various experimental conditions (type of NPs and cell lines), and cross-validation of the methods with other well-established analytical techniques.

## Figures and Tables

**Table 1:** Physicochemical properties of Ag NPs used in this study. Dispersion stability is the ratio of UV–vis absorbance values measured at 0 and 24 h. The standard deviations of 3 replicate measurements are given as confidence intervals.

NPs	Hydrodynamic Size (nm)				Surface Charge (mV)		Dispersion Stability	
	0 h	24 h	0 h	24 h	DI Water	RPMI-1640	DI Water	RPMI-1640
	DI Water	DI Water	RPMI-1640	RPMI-1640				
^(+)bPEI^Ag^40^	49.5 ± 0.02	49.0 ± 0.3	132.3 ± 1.0	165.5 ± 6.1	45.0 ± 0.7	−6.9 ± 0.4	0.998	0.769
^(+)bPEI^Ag^60^	68.4 ± 0.4	71.2 ± 0.2	118.2 ± 1.3	138.0 ± 0.7	33.8 ± 0.4	−11.3 ± 1.1	0.955	0.931
^(+)bPEI^Ag^80^	102.9 ± 0.7	102.1 ± 1.4	170.5 ± 1.8	180.3 ± 3.0	42.1 ± 0.7	−9.8 ± 0.7	0.973	0.808
^(+)bPEI^Ag^100^	113.1 ± 2.5	115.3 ± 1.3	213.6 ± 2.2	224.3 ± 3.3	63.8 ± 0.9	−11.7 ± 1.8	0.935	0.788
^(+)bPEI^Ag^200^	223.3 ± 4.7	225.5 ± 2.8	346.6 ± 3.8	342.7 ± 6.7	22.9 ± 1.6	−11.0 ± 1.1	0.897	0.684

**Table 2:** Applicability domain of regression equation correlating nSSC from flow cytometry with cellular uptake of three different nanoparticles.

Nanoparticle [Ref.]	Particle Size (nm)	Applicability Domain of Regression Equation		Regression Equation Type
		nSSC Intensity Range	Cellular Uptake Range	
Ag^bPEI^	40, 60, 80, 100, 200	~23	~80,000 NPs/cell	Linear
Au^bPEI^ and Au^Cit.^ [8]	40, 60, 80, 100	~1.6	~3500 NPs/cell	Linear
SiO_2_ [27]	30, 200, 300	~2.88	~14.5 ng/cell	Sigmoidal
